# Throwing Fracture of the Humerus in an Adolescent Patient Exposed to Isotretinoin: A Case Report of Successful Nonoperative Management

**DOI:** 10.7759/cureus.109035

**Published:** 2026-05-17

**Authors:** Adam N Fano, Daniel E Davis

**Affiliations:** 1 Department of Orthopaedic Surgery, Rothman Orthopaedic Institute, Philadelphia, USA

**Keywords:** baseball, functional bracing, isotretinoin, pitching, throwing fracture

## Abstract

Throwing fractures of the humerus are uncommon and typically occur in deconditioned adults. When they occur in adolescents, investigation into potential secondary causes is paramount.

Medication history may provide insight into potential secondary causes of humerus fracture and should be investigated. While it is unclear whether isotretinoin plays a clinically significant role in fracture risk in humans, it has been associated with alterations in bone metabolism and should remain a topic of further study. Nonoperative management with a functional brace continues to be a mainstay of treatment in the absence of surgical indications.

## Introduction

Throwing fractures of the humerus are seen less frequently than those related to direct trauma. The complex motions that occur at the shoulder and elbow during throwing, however, can lead to torsional forces on the humerus great enough to result in fracture. Typically, fractures in this setting occur during the late cocking and early acceleration phases of throwing where transitional motions result in the greatest amount of torsional force on the humerus [1,2]. Fractures of this nature are most commonly seen in recreational adult athletes who present with spiral fractures of the mid to distal humerus [1,3]. Much less commonly, this fracture may occur in the pediatric population, typically teenagers, with a spiral fracture of the mid to proximal humerus [2-4].

Given the rarity of throwing fractures in the pediatric population, investigation into secondary causes is particularly important. Contributing factors have been cited as disruption of regular practice, muscular fatigue, pain, inadequate skill level, and dysfunction/incoordination of antagonistic muscles [3]. Initial workup in any age group should include investigation into prodromal symptoms and medical history that may indicate an underlying stress fracture, low bone mineral density, or pathologic lesion [1,2,4,5]. Imaging should include a complete series of orthogonal radiographs as well as consideration of computed tomography (CT) scan, magnetic resonance imaging (MRI), or bone scan to investigate for abnormalities [4]. Closed, isolated, and non-pathologic pediatric humeral shaft fractures have historically been treated with nonoperative management [2,6], but there are reports demonstrating a recent shift toward operative intervention [7,8], with some studies reporting faster time to mobilization [9] and earlier return to sport [10].

The purpose of this report is to describe our experience with a throwing fracture of the humerus in an adolescent patient exposed to isotretinoin, successfully treated with nonoperative management. We suggest an emphasis on medication history as an important component of the initial evaluation and strong consideration of nonoperative management in the absence of contraindications.

## Case presentation

A 16-year-old left-hand-dominant male adolescent presented to the emergency room of our academic tertiary care hospital with left arm pain. He reported feeling a popping sensation and experiencing immediate pain in his left upper arm while throwing as a pitcher in his baseball game earlier that day. This event occurred mid-pitch. He was able to ambulate off the field and was brought directly to our emergency room by his mother for evaluation. On direct questioning, he reported pain in the left upper arm but denied pain elsewhere. He denied antecedent bony pain. He denied significant medical history and stated his only medication was oral isotretinoin he started for recalcitrant acne approximately one month prior. The patient had reported pitch speed ranging from ~85 to 90 mph.

**Figure 1:**
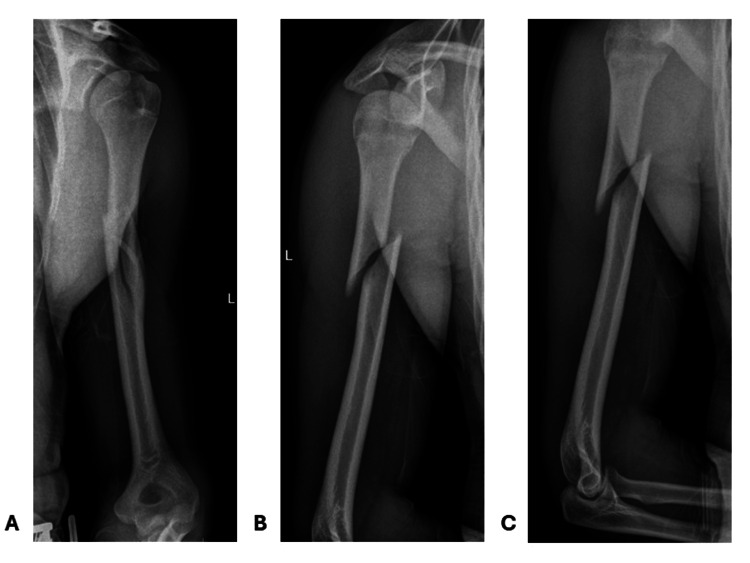
Anteroposterior (A) and lateral (B, C) radiographs from date of injury (10/21/2023)

On examination, there was swelling present about the upper arm but no obvious deformity. His skin was intact, and there was no prominent ecchymosis. Tenderness was elicited on palpation of the upper arm; there was no tenderness about the shoulder joint, elbow, forearm, wrist, or hand. Axillary, radial, median, and ulnar nerves were intact on motor and sensory testing, and radial pulse was palpable. Radiographs were obtained and revealed a spiral humeral shaft fracture located at the proximal to mid-shaft (Figure 1). He was placed into a splint and transitioned to a functional brace in the outpatient clinic.

**Figure 2:**
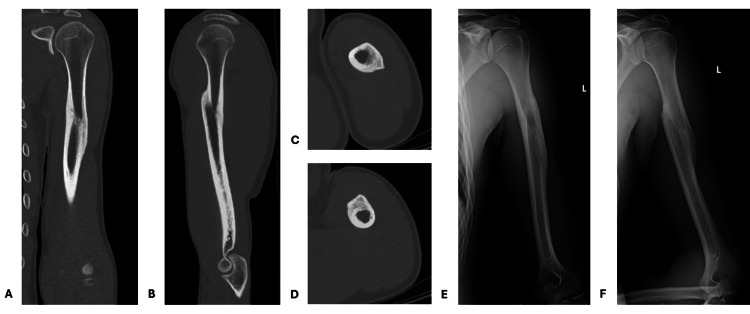
Select coronal (A), sagittal (B), and axial (C, D) cuts of computed tomography scan 7.5 months post-injury (6/7/2024). Anteroposterior (E) and lateral (F) radiographs one year post-injury (10/23/2024)

Both CT scan and MRI were completed to investigate for an underlying bone lesion and were negative. Elbow range of motion was started at two weeks, pendulum shoulder exercises were started at three weeks, and passive shoulder range of motion was started at four weeks. Active range of motion without resistance was started at six weeks. At this time, discussions were had regarding isotretinoin and fracture healing, with involvement of their dermatologist, and the family elected to stop the medication. Gentle strengthening exercises were started at eight weeks. At four months, there was near-complete osseous healing noted on X-ray, and an interval throwing program was initiated. A CT scan was performed at approximately 7.5 months post-injury to confirm complete healing of the fracture (Figure 2A-2D). He had achieved his pre-injury pitching speed at nine months post-injury and had surpassed this speed by one year (Figure 2E-2F).

## Discussion

Throwing fractures in the pediatric population are rare, but there have been select cases reported in the literature [2-4]. Most of these reports stress the importance of investigation into secondary causes which may relate to overtraining/undertraining [3] or underlying bone abnormality [1,2,4,5]. While treatment of pediatric humeral shaft fractures has historically been nonoperative [2,6], there have been reports demonstrating an increase in operative fixation [7,8]. Here, we describe an adolescent patient with a throwing fracture of the humerus in the setting of isotretinoin exposure and our experience with successful nonoperative management.

Interestingly, our patient had exposure to isotretinoin for about one month prior to injury. Isotretinoin is a derivative of vitamin A touted as a highly effective drug for recalcitrant acne [11]. However, several dangerous side effects are disclosed, the most well-known being teratogenicity leading to skeletal defects in the child [11]. Postnatally, exposure to vitamin A derivatives may result in the premature closure of lower extremity physes and a reduction in bone mineral density [11-14]. In animal models, hypervitaminosis A has been shown to result in weight loss, osteoporosis, and thinning of long bones [15]. Vitamin A derivatives seem to have the greatest effect on bone mineralization in a vitamin D-deficient setting [11,16,17]. It may be the interplay between vitamin A and vitamin D that is most important, and the current recommended daily amount of vitamin D, 600 IU for those aged less than 70 years, should be recommended to patients [18]. As prescribing of isotretinoin for recalcitrant acne increases [19,20], we stress the importance of investigating medication history when evaluating patients for atypical bony injury such as a throwing fracture. While it is unclear whether isotretinoin plays a clinically significant role in bone health and risk of fracture in humans, we suggest that medication history and its effect on fracture risk become a topic of further clinical and basic science research to determine its role.

Spiral fractures of the humeral shaft tend to respond well to nonoperative management options such as hanging arm casting, sling immobilization, splinting, and bracing due to the large surface area at the fracture site for the formation of bridging callus and the shoulder's inherent ability to compensate for malalignment, with the addition of high remodeling capacity in skeletally immature patients [7-9]. In line with these principles, treatment has been predominantly nonoperative [2,6], but recent reports have demonstrated an increased interest in operative fixation [7,8]. A 2017 report on humeral shaft fracture management in the adult population in the United States demonstrated a >10% increase in the utilization of open reduction and internal fixation (ORIF) from 2002 to 2011 [8]. A 2020 report out of Finland set out to investigate trends in the pediatric population, and they found a nearly 20% increased likelihood of operative treatment from 2001 to 2015 [7]. These observations are in light of select reports describing faster time to mobilization [9] and earlier return to sport [10] with operative treatment. In our experience, nonoperative management continues to be the mainstay of treatment in the absence of contraindications such as open fracture, vascular injury, brachial plexus injury, or polytrauma. Our patient went on to achieve an uncomplicated union and had achieved his pre-injury level of function by nine months post-injury.

## Conclusions

In addition to an investigation into prodromal symptoms, training practices, and medical history that may provide insight into potential secondary causes, we suggest that medication history is an important component of the initial evaluation of a patient presenting with a throwing fracture of the humerus. Nonoperative management with a functional brace continues to be a mainstay of treatment for humeral shaft fractures in the absence of surgical indications. With thorough questioning, the aid of diagnostic imaging, and discussions regarding operative and nonoperative management options, patients can be counseled properly and this entity treated successfully.
